# Two-Year Results of Injectable Matrix-Associated Autologous Chondrocyte Transplantation in the Hip Joint: Significant Improvement in Clinical and Radiological Assessment

**DOI:** 10.3390/jcm12175468

**Published:** 2023-08-23

**Authors:** Moritz Riedl, Henriette Bretschneider, Michael Dienst, Klaus-Peter Günther, Stefan Landgraeber, Jörg Schröder, Siegfried Trattnig, Stefan Fickert

**Affiliations:** 1 Department of Trauma Surgery, Regensburg University Medical Center, 93053 Regensburg, Germany; 2 University Centre for Orthopaedics and Trauma Surgery, University Hospital Carl Gustav Carus at Technische Universität Dresden, 01307 Dresden, Germany; 3 Orthopädische Chirurgie München, OCM Klinik GmbH, 81369 Munich, Germany; 4 Department of Orthopedic Surgery, Saarland University Medical Center, 66421 Homburg, Germanyfickert@sporthopaedicum.de (S.F.); 5 Department of Orthopedic Surgery, Klinikum Ernst von Bergmann Potsdam, 14467 Potsdam, Germany; 6 Christian Doppler Laboratory for Clinical Molecular MR Imaging (MOLIMA), Department of Biomedical Imaging and Image-Guided Therapy, Medical University of Vienna, 1090 Vienna, Austria; 7 High Field MR Centre, Department of Biomedical Imaging and Image-Guided Therapy, Medical University of Vienna, 1090 Vienna, Austria; 8 Sporthopaedicum Straubing Berlin Regensburg, 94315 Straubing, Germany

**Keywords:** hip-preserving surgery, hip arthroscopy, matrix-associated autologous chondrocyte transplantation, MACT, cartilage defect, femoroacetabular impingement

## Abstract

Purpose: Articular cartilage defects are a prevalent consequence of femoroacetabular impingement (FAI) in young active patients. In accordance with current guidelines, large chondral lesions of the hip joint over 2 cm^2^ are recommended to be treated with matrix-associated, autologous chondrocyte transplantation (MACT); however, the conditions in the hip joint are challenging for membrane-based MACT options. Injectable MACT products can solve this problem. The purpose of the trial was to assess clinical and radiological outcomes 24 months after injectable MACT of focal chondral lesions caused by FAI. Methods: We present data of 21 patients with focal cartilage defects of the hip [3.0 ± 1.4 cm^2^ (mean ± SD)], ICRS Grade III and IV caused by CAM-type impingement, who underwent arthroscopic MACT (NOVOCART^®^ Inject) and FAI correction. The outcome was evaluated with the patient-reported outcome instruments iHOT33 and EQ-5D-5L (index value and VAS), whilst graft morphology was assessed based on the MOCART score over a follow-up period of 24 months. Results: The iHOT33 score increased significantly from 52.9 ± 21.1 (mean ± SD) preoperatively to 85.8 ± 14.8 (mean ± SD; *p* < 0.0001) 24 months postoperatively. The EQ-5D-5L index value (*p* = 0.0004) and EQ-5D VAS (*p* = 0.0006) showed a statistically significant improvement as well. MRI evaluation after 24 months showed successful integration of the implant in all patients with a complete defect filling in 11 of 14 patients. Conclusions: Injectable MACT for the treatment of full-thickness chondral lesions of the hip joint due to FAI in combination with FAI correction improved symptoms, function, and quality of life in the treated cohort. Alongside the treatment of the underlying pathology by the FAI correction, the developed cartilage defect can be successfully repaired by MACT, which is of considerable clinical relevance.

## 1. Introduction

Articular chondral lesions represent a frequent and significant orthopedic concern in young athletic patients. In the hip joint, one of the primary causes of chondral damage is femoroacetabular impingement (FAI). This pathological condition arises when there is abnormal contact between the femur and the acetabulum of the pelvis during movement, resulting in repetitive friction and damage to the chondral tissue. Left untreated, FAI-related chondral damage can lead to progressive deterioration of the hip joint and finally to the development of hip osteoarthritis [1,2]. Due to the limited intrinsic self-regenerative capacity of the bradytrophic cartilage tissue in adults, there is an increasing need for efficient and consistent therapeutic options in the therapy of articular cartilage defects.

Over the past few decades, extensive research efforts have been devoted to developing methods for articular cartilage repair, aimed at alleviating pain, restoring joint function, and preventing the progression of degenerative joint diseases. Among the array of treatment options available, bone-marrow-stimulating techniques and autologous chondrocyte transplantation (ACT) became the most established procedures for addressing articular chondral lesions in the hip. However, their precise indications and effectiveness continue to be subjects of ongoing debate within the orthopedic community [3]. The German Society for Orthopedic and Trauma (DGOU) working group “clinical tissue regeneration” and the Hip Committee of the Society for Arthroscopy and Joint Surgery (AGA) have issued recommendations in favor of matrix-associated ACT (MACT) as a first-line treatment for full-thickness hip chondral defects larger than 2 cm^2^, whereby less invasive forms of MACT (e.g., injectable chondrocyte transplants) should be favored in the hip due to the so far low complication rates and predominantly favorable clinical results [4].

As conventional matrix-based ACT products require challenging fixation procedures within the defect area, these techniques are not applicable for minimally invasive use in the hip joint due to its anatomical conditions with limited joint space in the central compartment and the osseous and labral containment.

The injectable, in situ crosslinking MACT product NOVOCART^®^ Inject overcomes this limitation, since it can be implanted arthroscopically into the lesion site without additional fixation. This study analyses the clinical outcome and MRI-assessed morphology 24 months after injectable MACT for the treatment of full-thickness cartilage lesions in the hip caused by FAI. As the understanding of cartilage repair mechanisms continues to evolve, this research is poised to make a meaningful impact on the lives of individuals seeking effective solutions for the restoration of joint health and function.

## 2. Materials and Methods

This trial is a prospective, multicenter case series, with patients treated in three centers from January 2015 to November 2015, and is listed on ClinicalTrials.gov (NCT02179346). The study was approved by the local institutional review board (#EK 48022014).

### 2.1. Study Design

In this study, patients aged between 18 and 60 years with focal full-thickness cartilage defects of the hip with ICRS grade III or IV caused by CAM-type FAI were to be included. Eligible study patients had provided written informed consent prior to any study-specific measures, and the local data protection law was observed throughout the course of the study and data analysis. Requirements for MACT application were a defect size between 1.5 and 10 cm^2^, stable defect edges, and intact surrounding cartilage matrix, subchondral bone lamella, and corresponding articular surface [5]. Exclusion criteria comprised corresponding cartilage defects, more than two chondral lesions, radiographic evidence of osteoarthritis higher than grade 1 according to Kellgren and Lawrence, cartilage defects in both lower extremities, deep osseus lesions in the defect area, presence of rheumatoid, infectious, or parainfectious arthritis, skin injury to the limb to be operated on, acute abuse of medications, drugs, or alcohol, known allergy to components of NOVOCART^®^ Inject, or severe internal diseases (infectious, cardiovascular, endocrine, metabolic, autoimmune, neoplastic).

For preoperative diagnostics, a clinical examination of the hips, X-rays with standardized supine anterior–posterior and cross-table views, and magnetic resonance imaging resp. arthro-MRI including radial planes [6,7] were performed. Clinical results were evaluated by the two general patient-reported outcome (PRO) instruments, the EQ-5D-5L questionnaire (standardized health-related quality-of-life measure for the assessment of an individual’s health status across five dimensions: pain/discomfort, mobility, usual activities, anxiety/depression, mobility, and self-care) summarized in a global index score, and the EQ-5D VAS (visual analogue scale), as well as the hip specific clinical outcome measurement device iHOT33 (assessment of hip specific pain and functional parameters of daily living and sports) [8,9]. The patient-reported outcome and MR images were obtained before index arthroscopy (preoperative) and 6, 12, and 24 months after MACT.

### 2.2. Surgical Technique

In this study, patients with focal acetabular chondral lesions underwent a two-step treatment using either arthroscopic or arthroscopically assisted autologous chondrocyte transplantation (MACT). The initial procedure involved analyzing and grading the defect area with the focal chondral lesion, following the grading system of the International Cartilage Repair Society (ICRS). For arthroscopy, patients were positioned on a traction table and joint distraction of 6–8 mm was applied. Once the indication for treatment was confirmed, cartilage biopsies were harvested from non-weight-bearing areas around the joint’s head–neck junction. These biopsies, along with a 10 mL sample of the patient’s blood, were then sent to the manufacturer (TETEC Tissue Engineering Technologies AG, Reutlingen, Germany). Concomitant corrective surgeries, such as addressing the head–neck offset and labrum repair, were performed either during the first or the second surgery, depending on the patient’s specific needs. The second intervention, which took place approximately three to four weeks after the initial arthroscopy, when manufacturing was complete and NOVOCART^®^ Inject was delivered by the provider, involved applying MACT, either through an arthroscopic approach or through a minimally invasive (arthroscopically assisted) limited anterior approach. After initial debridement of the lesion site, the chondral defect was carefully filled with NOVOCART^®^ Inject up to the level of the surrounding, perilesional cartilage. Using a dual chamber syringe, the autologous cell suspension was injected, together with a crosslinking component, into the prepared defect [10,11]. The resulting crosslinked bioresorbable albumin/hyaluronan hydrogel then kept the chondrocytes in the desired location without the need for additional fixation.

### 2.3. Rehabilitation

All patients completed a standardized postoperative rehabilitation program. Patients were prescribed partial weight bearing with 15 kg of body weight for a duration of 6 weeks followed by a gradual increase in load, with 10–20 kg added each week, until patients were able to resume full weight bearing. Return to competitive sports was permitted only after a minimum period of 9–12 months following the operation, allowing ample time for proper healing and recovery. Continuous passive motion (CPM) therapy was applied for 4 weeks with a minimum requirement of 6 h of therapy a day. Postoperative aftercare included common measures to prevent complications. To avoid formation of heterotopic ossifications, patients were prescribed oral medication with a nonsteroidal anti-inflammatory drug, diclofenac, at a dosage of 3 × 50 mg/day for 2 weeks postoperatively. Additionally, to prevent deep vein thrombosis (DVT), anticoagulation therapy with low-molecular-weight heparin was administered until full weight-bearing capability was reached.

### 2.4. Manufacturing Process of NOVOCART^®^ Inject

After harvesting, the cartilage biopsies were sent to the manufacturing facility (TETEC AG, Reutlingen, Germany). Chondrocytes were manufactured according to a standardized and approved protocol under the guidelines of Good Manufacturing Practice (GMP). Cells were isolated from the biopsies and expanded for three to four weeks in cell culture depending on the cell growth and the date of implantation. The cells are not passaged to minimize de-differentiation. As a last step, the cell suspension is supplemented by modified human serum albumin (MAHSA) and sodium hyaluronate. The second component of NOVOCART^®^ Inject acts as a MAHSA crosslinker, to in situ form an albumin/hyaluronan hydrogel. Extensive and validated quality control analyses during manufacturing and at the time of product release (e.g., regarding cell number, cell viability, and quantitative expression of marker genes) assured that the product was always within controlled specification limits.

### 2.5. Magnetic Resonance Imaging and Evaluation

For MRI of the hip joints, a 1.5 resp. 3.0 T MR Scanner was used. Examinations of all patients were performed after 6, 12, and 24 months including a sagittal proton-density (PD) weighted turbo-spin-echo (TSE) sequence with frequency-selective fat suppression (FS), a coronal PD-TSE sequence with FS, a coronal high-resolution PD-TSE, and a three-dimensional T1 water-excited gradient-echo sequence. By magnetic resonance observation of cartilage repair tissue grading scale (MOCART), quality and maturation of the repair tissue were evaluated based on the degree of defect filling, integration to adjacent cartilage, and structure as well as signal intensity of repair tissue [12,13]. The original score includes a signal intensity evaluation using the T1 weighted 3D-GE-FS sequence with the categories “isointense” (15 points), “moderately hypointense” (5 points), and “markedly hypointense” (0 points). In this study, signal intensity evaluation using the 3D-GE-FS sequence was not available. Thus, the MOCART score ranges from 0 to 85 instead of 100 score points.

### 2.6. Statistical Analysis

Statistical analysis was carried out using the software package SAS, Version 9.4., SAS Institute Inc. Cary, NC, USA. Continuous data are presented as mean and standard deviation (SD). Score changes to preoperative value were analyzed on quantified data using two-sided paired t-tests. A significance level of 0.05 was used to determine statistical significance. To explore potential correlations between the MOCART score and clinical outcomes, Pearson correlation coefficients were calculated. All methods had been previously described separately [14].

## 3. Results

**Figure 1:**
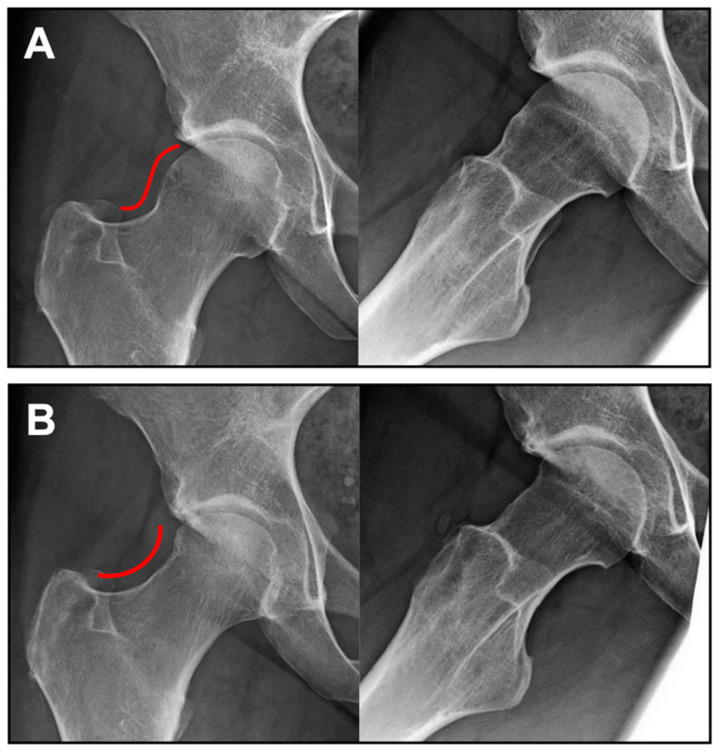
Anterior–posterior and axial X-ray imaging preoperative (**A**) with present CAM deformity and 6 months postoperative (**B**) after head–neck recontouring in combination with MACT. Change in the head–neck offset is demonstrated by the red line.

Twenty-one patients aged between 20 and 53 years were included in the study. All patients were diagnosed with full-thickness cartilage defects, ICRS grade III–IV. Most of the patients (19 patients, 90.5%) had one defect, 2 patients (9.5%) had two defects. The mean total defect size was 3.0 cm^2^ (range: 1.5–8.0 cm^2^). All patients underwent a concomitant osteochondroplasty of the head–neck region for correction of the underlying CAM deformity (in 14 patients arthroscopically and in 7 patients through mini-open limited anterior approach; Figure 1). Labrum reconstruction was necessary in 13 patients (61.9%). An additional surgical intervention on the contralateral hip after the treatment with NOVOCART^®^ Inject was documented for six patients (28.6%). The patients stayed in hospital for 4.2 ± 2.9 days (mean ± SD) after implantation of NOVOCART^®^ Inject. Table 1 shows a detailed summary of the population’s demographic data and baseline characteristics.

During the first observation period of 12 months post-surgery, two serious adverse events (SAEs) (bacterial arthritis, persistent arthralgia) and one nonserious adverse event (AE) (superficial wound healing disturbance) occurred in 3 out of 21 patients as described in detail in the previous publication [14]. These events were considered surgery-related and not related to the ACT product. A third SAE was assessed as not associated with the NOVOCART^®^ Inject procedure as it was a surgical treatment of the contralateral (untreated) hip joint related to the underlying disease.

Complete follow-up of patient-reported outcomes could be obtained until month 12, whereas on month 24, four patients were lost to follow-up.

**Figure 2:**
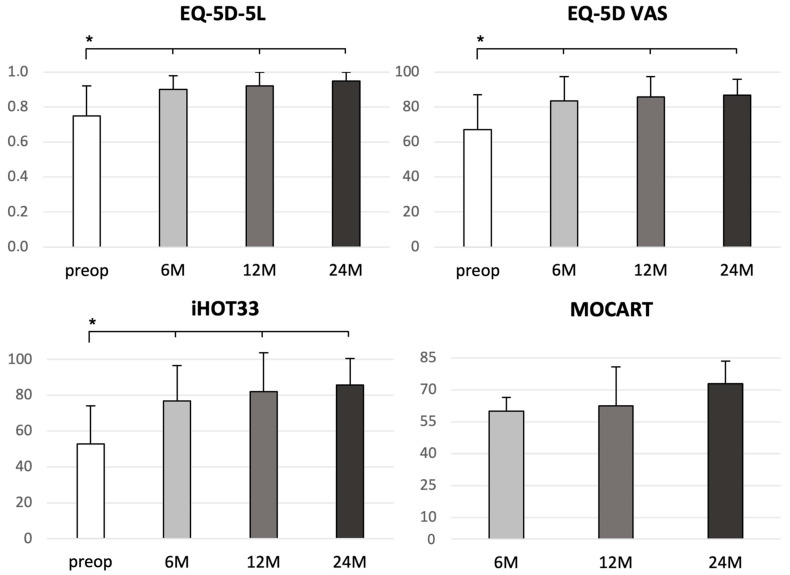
Outcome evaluation of iHOT 33, EQ-5D-5L, EQ-5D VAS, and MOCART preoperative and at 6-, 12-, and 24-months follow-up. Whiskers indicate standard deviation. Asterisks mark statistically significant differences.

The iHOT33 score increased significantly after 6 [23.3 ± 26.3 (mean ± SD); *p* = 0.0008], 12 [29.2 ± 30.3 (mean ± SD); *p* = 0.0005], and 24 months [33.6 ± 25.3 (mean ± SD); *p* ≤ 0.0001] compared to the preoperative iHOT33 score (additional absolute values: Table 2; Figure 2). The minimal clinically important difference (MCID) for the iHOT33 was determined to be 6 points [8]. The responder rates (i.e., the proportions of patients achieving MCID) at months 6, 12, and 24 were 75.0%, 78.9%, and 87.5%, respectively. There were some clues that patients without subsequent contralateral hip surgery had experienced slightly stronger improvements in the iHOT33 outcomes than vice versa, but the sample sizes were too small to allow generalizable conclusions.

The study cohort showed significant improvements in quality of life following MACT of the hip, as indicated by both the EQ-5D-5L index value and EQ-5D VAS (additional absolute values are presented in Table 2). When compared to the preoperative scores, the changes in EQ-5D-5L and EQ-5D VAS were statistically significant at 6 months [EQ-5D-5L: 0.15 ± 0.17 (mean ± SD); *p* = 0.0011; EQ-5D VAS: 16.5 ± 18.8 (mean ± SD); *p* = 0.0006], 12 months [EQ-5D-5L: 0.16 ± 0.19 (mean ± SD); *p* = 0.0007; EQ-5D VAS: 19.0 ± 19.7 (mean ± SD); *p* = 0.0003], and 24 months [EQ-5D-5L: 0.17 ± 0.16 (mean ± SD); *p* = 0.0004; EQ-5D VAS: 18.2 ± 17.6 (mean ± SD); *p* = 0.0006] (Table 2; Figure 2). These findings demonstrate the positive and sustainable impact of MACT on the overall quality of life of the patients throughout the study period.

**Figure 3:**
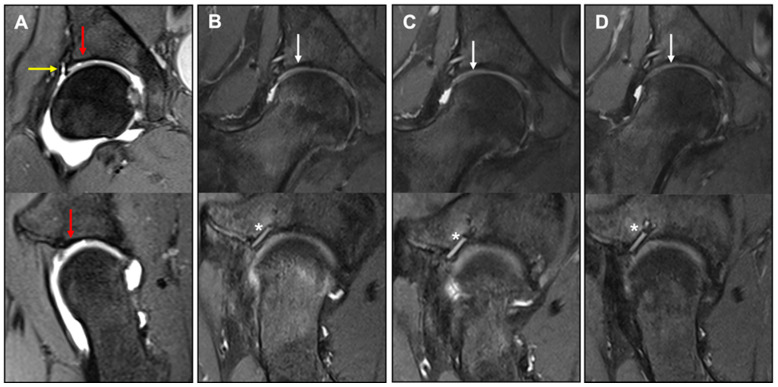
Coronar and sagittal preoperative arthro-MRI (**A**) with acetabular cartilage defect (red arrow) and labral tear (yellow arrow) and coronar and sagittal PD-TSE FS MRI 6 months (**B**), 12 months (**C**), and 24 months (**D**) postoperative with anker refixation of the labrum (asterisk) and cartilage repair tissue (white arrow) in the defect side with complete integration and a complete filling of the defect.

MR images were assessed using the MOCART score 6, 12, and 24 months after MACT (Figure 3). Since no 3D-GE-FS measurements were performed in this study, the maximum achievable score value was 85 points instead of 100 points. In 2 of the 21 study patients, MRI measurements were uninterpretable due to metalware artifacts and missing fast-spin-echo, respectively. In 5 further patients, the assessments at month 24 were not performed. Consequently, postoperative MRIs of 20 and 14 patients were available for follow-up at 12 and 24 months, respectively.

A complete filling of the defect was found in 11 patients (78.6%) at 24 months. In all patients, a complete integration of the implant at the border zone was achieved without visible delamination or demarcation between repair tissue and surrounding cartilage 24 months after MACT. The surface of the repair tissue was intact and smooth without fibrillation in 11 patients (78.6%) and the subchondral bone was uninjured in 9 patients (64.3%). In 8 patients (57.1%), an isointense signal of the repair tissue was detected on TSE sequence compared to healthy cartilage. The total MOCART score was 60 ± 6.5 (mean ± SD) at 6 months, 62.5 ± 18.3 (mean ± SD) at 12 months follow-up, and 72.9 ± 10.7 (mean ± SD) at 24 months postoperatively. Correlation analyses with the clinical variables (iHOT33, EQ-5D index and VAS) did not suggest clinically meaningful correlations between MOCART score and patient-reported clinical outcomes.

## 4. Discussion

The main objective of this research study was to comprehensively investigate the clinical and radiological outcomes following the application of NOVOCART^®^ Inject in patients with full-thickness cartilage defects of the hip joint. The study sought to elucidate the potential benefits and efficacy of this innovative treatment approach, involving injectable MACT in conjunction with arthroscopic femoroacetabular impingement correction. Significantly, the results of the study revealed a remarkable improvement in symptoms, functional outcomes, and overall quality of life among the patients who underwent this combined procedure. The most noteworthy radiological finding was the achievement of complete defect filling in the majority of patients, as observed by MRI. This indicated successful cartilage repair and regeneration, contributing to the observed clinical improvements in patient-reported outcomes. Throughout the follow-up periods of 6, 12, and 24 months after surgery, there were consistent and statistically significant increases in the iHOT33, the EQ-5D-5L index value, and EQ-5D VAS compared to the baseline measurements, with the most substantial progress evident by the 6th month and persisting up to 24 months. This suggests the enduring and positive effect of the NOVOCART^®^ Inject procedure on patients’ overall well-being, quality of life, and hip-specific clinical outcome.

To assess MRI images with regard to the condition of cartilage repair tissue, the MOCART score was applied 6, 12, and 24 months postoperatively. The MOCART score is an established classification system for the evaluation of cartilage repair [15]. The percentages of patients with complete defect filling and complete graft integration were highest at 24 months (78.6% and 100%, respectively). This is consistent with current evidence on the knee joint indicating that graft maturation after ACT needs at least 12 to 24 months before cartilage regeneration is completed [16]. Complete integration of the implant at the defect’s margin area without visible debonding or demarcation between repair tissue and surrounding cartilage in all patients, as well as intact cartilage surface at 24 months post intervention in most of our patients, are promising results. Thus, the clinical improvement seen in the iHOT33 and EQ-5D-5L variables was accompanied by radiological improvement in the MOCART score up to 24 months. However, correlation analyses did not reveal clinically meaningful correlations between clinical and radiological outcomes. This is in accordance with a meta-analysis of 32 studies including MRI assessment of cartilage implants in the knee by de Windt et al., who identified a correlation between the MOCART score and clinical results in only 28% of the studies [17]. Lazik et al. also did not find a correlation between MOCART score and clinical outcome (NAHS) after MACT in the hip [12]. Nonetheless, it should be noted that positive MRI results at earlier time points could potentially serve as predictive indicators for long-term outcomes. This was demonstrated by McCarthy and co-workers on the knee joint, where MRI parameters evaluated at 12 months showed a significant correlation with sustained, favorable clinical long-term results [18]. Accordingly, correlations between radiological and clinical results were not necessarily to be expected in this study, particularly since the MOCART score, which is often compared with clinical outcome, was initially developed as a standardized imaging evaluation system for cartilage tissue, and not for prediction of clinical outcome. Instead, functional (biochemical) MRI such as delayed gadolinium-enhanced MRI of cartilage (dGEMRIC), quantitative T1rho, T2 mapping, or chemical exchange saturation transfer on glucosaminoglycans (GagCest) might be more capable of assessing cartilage repair with regard to the clinical outcome [19,20]. However, availability and applicability of these techniques are currently limited, and the interpretation is challenging.

NOVOCART^®^ Inject is especially suited for application in the hip joint compared to membrane-based MACT products, as their fixation is especially challenging in the limited hip joint space, particularly if arthroscopic application is to be performed. For this purpose, the solid cell-seeded biomaterial must be rolled up and introduced into the defect via a portal, similar to the AMIC procedure [21]. Under these conditions, significant biomechanical stresses may occur, leading to damage and death of chondrocytes in the biomaterial [22] and consequently to significantly worse clinical results [23]. In addition, suturing of articular cartilage for implant fixation was shown to induce severe local damage and to be related to early stages of osteoarthritis [24]. As an injectable and self-adhering implant, NOVOCART^®^ Inject overcomes these disadvantages and opens new opportunities for minimally invasive cartilage therapy in narrow joint spaces such as the hip joint.

In their current treatment guidelines for the reconstruction of full-thickness cartilage defects of the hip, the DGOU group “Clinical Tissue Regeneration” and the Hip Committee of the AGA recommend the preferential use of injectable products in patients that meet the indications for a MACT procedure in the hip for focal full-thickness chondral defects larger than 1.5–2 cm^2^ without signs of osteoarthritis [4]. As NOVOCART^®^ Inject is not the only injectable MACT product on the market, there are alternatives such as autologous chondrocyte spheroids. The feasibility of arthroscopic MACT for full-thickness cartilage lesions could be confirmed for both options in previous studies [25,26,27,28,29]. No significant differences were reported concerning clinical outcome between the two MACT procedures [28]. However, NOVOCART^®^ Inject might provide a potential advantage in surgical handling due to the remarkable bonding capacity of the hydrogel for which in situ crosslinking is achieved within 1 to 3 minutes. Despite the mentioned advantages of injectable MACT products, the procedure is a challenging intervention even for experienced arthroscopists. The orientation of the acetabulum as well as the often antero-laterally localized cartilage lesions make it necessary to work upside-down, perpendicular to the skin surface. Depending on the labrum thickness, there may be a further reduction in the available work area, a problem which may also apply to NOVOCART^®^ Inject. The use of NOVOCART^®^ Inject should therefore only take place after careful training of the surgeon. However, if the technique of defect preparation and hydrogel application has been sufficiently trained, the use of NOVOCART^®^ Inject is usually possible even under these difficult conditions.

The evidence for the therapy of large full-thickness cartilage defects in the hip joint still needs improvement. However, there is consensus that minimally invasive surgical procedures are preferable over open surgery [4,25,26,27,28,29,30,31].

During the observation period, two serious adverse events (bacterial arthritis, persistent arthralgia) as well as one nonserious adverse event (superficial wound healing disturbance) were recorded. All of them are common postoperative complications in hip surgery related to the underlying pathology. Therefore, these incidents were not ascribed to the NOVOCART^®^ Inject treatment itself. Nevertheless, they were associated with the graft implantation surgery (bacterial arthritis and persistent arthralgia) or chondrocytes biopsy (superficial wound healing disturbance) in the context of the study.

The following limitations of the present study have to be considered. The study has no control group, which allows no comparison to other cartilage therapies. The number of included patients (*n* = 21) was quite low, just like in other MACT studies reporting results for groups of 6–30 patients [25,27,29,30]. The restriction to cartilage defects caused by FAI may be a selection bias and does not allow for transfer of these findings to patients with other mechanic disorders, although FAI is probably the main cause for localized cartilage lesions in the hip joint. Finally, in addition to the cartilage therapy, concomitant corrective surgeries were performed to eliminate the causative problem (e.g., labral refixation, femoroplasty, and acetabuloplasty). Therefore, it is not possible to distinguish the extent to which clinical improvement is due to cartilage repair or to the concomitant corrective surgery.

## 5. Conclusions

Based on the current study results, the treatment of full-thickness cartilage defects of the hip joint using injectable MACT with NOVOCART^®^ Inject was shown to be feasible and safe in a real-world setting. Injectable MACT in combination with arthroscopic FAI correction significantly improved symptoms, functional outcome, and quality of life in patients with chondral lesions of the hip joint caused by FAI. The effectiveness of NOVOCART^®^ Inject was evaluated with the patient-reported outcome instruments iHOT33 and EQ-5D-5L (index value and VAS), whilst graft maturation was assessed based on the MOCART score. These instruments consistently indicated significant and clinically meaningful improvements up to 24 months postoperatively. The majority of patients were found to have a complete defect filling by MRI. However, further randomized controlled trials with larger patient numbers and comparison to other treatment options have to confirm the results in long-term follow-up.

## Figures and Tables

**Table 1:** Demographic data and baseline characteristics of the study patients (*n* = 21).

Sex		
Female	*n* (%)	4 (19.1)
Male	*n* (%)	17 (81.0)
Age (years)	Mean ± SD	32.3 ± 10.0
BMI (kg/m^2^)	Mean ± SD	25.5 ± 3.6
Smoking status		
Yes	*n* (%)	5 (23.8)
No	*n* (%)	16 (76.2)
Defect side		
Right hip	*n* (%)	14 (66.7)
Left hip	*n* (%)	7 (33.3)
Number of lesions		
1	*n* (%)	19 (90.5)
2	*n* (%)	2 (9.5)
Total defect size (cm^2^)	Mean ± SD	3.0 ± 1.4
	Range	1.5–8.0
Defect localization		
Acetabulum only	*n* (%)	19 (90.5)
Acetabulum + head	*n* (%)	2 (9.5)
ICRS grading		
Grade III	*n* (%)	17 (81.0)
Grade IV	*n* (%)	4 (19.1)
Previous hip surgery		
Yes	*n* (%)	3 (14.3)
No	*n* (%)	18 (85.7)

**Table 2:** Patient-reported outcome including iHOT 33, EQ-5D-5L, and EQ-5D VAS at preoperative stage and at 6-, 12-, and 24-months follow-up [mean ± standard deviation (SD)].

	Preoperative		6 Month Follow-Up		12 Month Follow-Up		24 Month Follow-Up	
	Mean	SD	Mean	SD	Mean	SD	Mean	SD
iHOT 33	52.9	21.1	76.8	19.8	82.0	21.9	85.8	14.8
EQ-5D-5L	0.75	0.17	0.90	0.08	0.92	0.08	0.95	0.05
EQ-5D VAS	67.0	20.1	83.5	14.0	85.9	11.6	86.7	9.3

## Data Availability

The data presented in this study are available on request from the corresponding author.
